# Contribution of Ultra-Fine Bubbles to Promoting Effect on Propane Hydrate Formation

**DOI:** 10.3389/fchem.2020.00480

**Published:** 2020-06-05

**Authors:** Tsutomu Uchida, Hiroshi Miyoshi, Ren Sugibuchi, Akio Suzuta, Kenji Yamazaki, Kazutoshi Gohara

**Affiliations:** ^1^Faculty of Engineering, Hokkaido University, Sapporo, Japan; ^2^Graduate School of Engineering, Hokkaido University, Sapporo, Japan

**Keywords:** nanobubble, stability, number density, memory effect, propane, induction time

## Abstract

To investigate experimentally how ultra-fine bubbles (UFBs) may promote hydrate formation, we examined the formation of propane (C_3_H_8_) hydrate from UFB-infused water solution using two preparation methods. In one method, we used C_3_H_8_-hydrate dissociated water, and in the other, C_3_H_8_-UFB-included water prepared with a generator. In both solutions, the initial conditions had a UFB number density of up to 10^9^ mL^−1^. This number density decreased by only about a half when stored at room temperature for 2 days, indicating that enough amount of UFBs were stably present at least during the formation experiments. Compared to the case without UFBs, the nucleation probabilities within 50 h were ~1.3 times higher with the UFBs, and the induction times, the time period required for the bulk hydrate formation, were significantly shortened. These results confirmed that UFB-containing water promotes C_3_H_8_-hydrate formation. Combined with the UFB-stability experiments, we conclude that a high number density of UFBs in water contributes to the hydrate promoting effect. Also, consistent with previous research, the present study on C_3_H_8_ hydrates showed that the promoting effect would occur even in water that had not experienced any hydrate structures. Applying these findings to the debate over the promoting (or “memory”) effect of gas hydrates, we argue that the gas dissolution hypothesis is the more likely explanation for the effect.

## Introduction

Gas hydrates that exist below the deep sea floor are both an unconventional natural gas resource and a potential source of greenhouse gas. In addition, gas-hydrate formation can be a nuisance when it starts to plug gas pipelines in cold regions. Such interests have stimulated much research and development on gas hydrates (Kvenvolden, 1988; Sloan, 2004; Sloan and Koh, 2007; Masuda et al., 2016). For example, with the gas pipeline issue, research has focused on suppressing the formation and growth of gas hydrate. However, as the hydrate form contains gas at relatively high density, gas hydrate is regarded as a promising medium for transporting and storing the gas (Gudmundsson and Borrehaug, 1996; Ida and Kohda, 2004; Horiguchi et al., 2011; Mimachi et al., 2014; Takeya et al., 2018).

Gas hydrate is formed by a reaction between water and the guest gas at low temperatures and high pressures. But the nucleation of gas-hydrate crystals requires a relatively large supercooling (or super-saturation). Such conditions necessitate additional energy for gas-hydrate formation and make it difficult to control the formation process. Thus, a key research goal is to find more efficient ways to form gas hydrates.

Propane (C_3_H_8_) is the main component of LPG and a component of natural gas. Its solubility in water (about 2.7 × 10^−5^ in mole fraction at 293.2 K; (The Chemical Society of Japan, 2004)) is similar to methane (CH_4_). The formation of C_3_H_8_ hydrate via the reaction between C_3_H_8_ gas and pure water is difficult (Christiansen and Sloan, 1994; Giavarini et al., 2003). This difficulty has been understood as a consequence of the labile-cluster nucleation hypothesis, in particular, a difficulty in forming hexakaidecahedral (5^12^6^4^) cavities (Christiansen and Sloan, 1994).

At present, the “memory effect” is the most promising way to increase the efficiency of forming gas hydrate (Ripmeester and Alavi, 2016). Another way to promote C_3_H_8_-hydrate formation is by using “ice-melting water,” which is water from just-melted ice Giavarini et al. (2003). Ida and Kohda (2004) investigated several such methods, arguing that the micro-bubble method was the most promising way. The mechanism by which this method works was argued to be the increase of gas-liquid interface. Zeng et al. (2006) confirmed the memory effect of C_3_H_8_ hydrate when they used the C_3_H_8_-hydrate melt water although they aimed to investigate the inhibition effect of anti-freeze proteins on the C_3_H_8_-hydrate formation.

The memory effect is a phenomenon in which once a formed crystal is dissociated into gas and water, and then reformed, the crystallization occurs with lower supercooling or supersaturation than when the crystal was initially formed. The mechanism is still under debate, and several hypotheses have been proposed. One hypothesis is the “water structuring hypothesis” that the fragments of hydrate-lattice structure remains in the dissociated water (Hwang et al., 1990; Parent and Bishnoi, 1996; Ohmura et al., 2003; Buchanan et al., 2005; Sloan and Koh, 2007; Sefidroodi et al., 2013). This is consistent with the concept that water has a dynamic structure, so it is considered to be a promising hypothesis. However, the existence of such “fragments” has not been established.

Another hypothesis is the “gas dissolution hypothesis” that comes from the requirement of a sufficient concentration of guest molecules in the liquid phase for hydrate to form (Rodger, 2000). Most guest molecules are hydrophobic, with relatively low solubility in water. In the crystalline gas hydrate, the gas concentration is hundreds of times its solubility in water, thus when the hydrate grows, a large amount of guest molecules must be supplied from the gas phase. For example, the mole fraction of C_3_H_8_ over H_2_O in the hydrate structure is estimated to be about two thousand times that of the C_3_H_8_ solubility in water. This difficulty of acquiring enough guest molecules is considered to be a major barrier to crystallization. Uchida et al. (2016a,b); Uchida et al. (2017, 2020) demonstrated experimentally the presence of ultra-fine bubbles (UFBs) in hydrate-dissociated water. They argued that the UFBs are a source of guest molecules to the liquid phase, and they suggested that these UFBs produce the memory effect via the gas dissolution hypothesis.

UFBs are small gas bubbles <1 μm (ISO 20408-1:2017, 2017). They have unique properties such as low buoyancy, high internal pressure, and a low rate of coalescence due to repulsive forces from their negative surface charges (ζ-potential) (Takahashi, 2005; Seddon et al., 2012; Oshita and Uchida, 2013). These properties allow UFBs to remain in the liquid for a long time. Usually, UFB-containing water is prepared with a fine-bubble generator. Our previous studies (Uchida et al., 2016a,b, 2017, 2020) have confirmed that gas hydrate dissociation produces a high concentration of UFBs in water. This phenomenon is also supported by molecular dynamic simulations (Yagasaki et al., 2014; Bagherzadeh et al., 2015).

The relationship between UFBs and the memory effect has been studied using CH_4_, ethane (C_2_H_6_), and carbon dioxide (CO_2_) hydrates (Uchida et al., 2016a,b, 2017, 2020). All of these hydrates have the same sI (structure-I) hydrate. Here we ask whether the UFBs have the same role in the memory effect of the sII (structure-II) hydrate by studying the effect experimentally using C_3_H_8_ gas. As UFBs used in the present study were much smaller than micro bubbles, our approach differs from the micro-bubble method proposed by Ida and Kohda (2004). Therefore, we also investigated the stability of C_3_H_8_-UFBs by their number density change with storage time at room temperature.

## Experimental Methods

### Materials and UFB Measurements

As in our previous studies (Uchida et al., 2016b, 2020), three liquid samples were used for the experiments: pure water, C_3_H_8_-hydrate dissociated water, and C_3_H_8_-UFB-included water. Pure water here means ion-exchanged distilled water of resistivity about 15 MΩ cm. The C_3_H_8_-hydrate dissociated water was prepared by dissolving about 2.5 g of C_3_H_8_-hydrate crystal in about 50 mL of pure water at about 293 K. The source crystal for this sample was retrieved from our reaction vessel at about 200 K. The C_3_H_8_-UFB-included water was prepared with a micro-bubble generator (Aura Tec, Fukuoka, Japan, type OM4-MDG-045) by supplying C_3_H_8_ gas (99% in purity, Hokkaido Air Water, Hokkaido, Japan) at 0.25 MPa into 1 L of pure water maintained at 293 K by immersing the water-filled beaker into the temperature-controlled bath (Otsuka Electronics, Osaka, Japan, type NM-454L). To obtain sufficient UFBs, the circulating time was set for 1 h. These liquids were used for the C_3_H_8_-hydrate formation test more than 1 h after the complete disappearance of micro- or macroscopic bubbles. The pH value was measured with a pH sensor (Sato Keiryoki, Tokyo, Japan, type SK-620PHII).

The number and size distributions of UFBs in the solution were measured by both laser-light scattering (LS) and by freeze-fractured replica observation via transmission electron microscope (FFT). In the LS technique, an Ar-ion laser (Omnichrome, CA, USA, type 543-150 GS, λ = 514.5 nm, 5 mW) light was introduced into an optical glass cell (Toshinriko, Tokyo, Japan, type PSK-3: about 1 cm^3^) in which each liquid sample had been dispensed. The 90-degree light scattering image was recorded by CCD camera (Watec, Yamagata, Japan, type WAT-232S) from which we counted the bright spots in a unit volume (using Image J software). The average number density was estimated from 16 images for each sample. Preliminary experiments have confirmed that this method can measure UFBs with a diameter of larger than 300 nm and with the number density more than 10^6^ mL^−1^ (Uchida et al., 2020).

The FFT method we used is described in detail in Uchida et al. (2016a,b,c); Uchida et al. (2020), so we describe it only briefly here. A small amount (<10 μL) of liquid sample was quickly frozen by immersing it into liquid nitrogen. The frozen sample was then set in the replication system (JEOL, Tokyo, Japan, type JFD-9010) and fractured under low temperature (about 150 K) and high vacuum (<10^−4^ Pa) conditions to form a freshly fractured surface. On this surface, both platinum and carbon were deposited to form a thin film that replicates the roughness of the fractured surface. After transferring to a Cu-grid having 43 μm × 43 μm opening, we observed the fractured surface using a high-resolution transmission electron microscope (TEM: JEOL, JEM-2010, at 200 kV accelerating voltage). An imaging plate (Fujifilm, Tokyo, Japan, type FDL-UR-V) was used for acquiring the observed image. This method allows us to observe UFBs, and distinguish them from impurities (the former is a hemispherical hollow, the latter sticks up). To obtain the average value of the UFB distribution, we measured at least three independent replica-film samples for a specified liquid sample.

To overcome the limitation of the FFT measurement and to cover the wider size-ranged UFBs, we combine another measurement method. For observing the smaller UFBs, we used the commercially available particle tracking analysis (PTA) method (Quantum Design Japan, Tokyo, Japan, type NS500, λ = 635 nm). This system allowed us to obtain the particle size distributions and the average number density of UFBs having diameters of about 20–300 nm. The averaged values for the UFB distribution were estimated from at least six measurements for a specified liquid sample.

With the above methods, we measured the size and density of UFBs immediately after preparing the samples by storing the liquid sample in glass bottles (about 6.5 mL, without head space) at room temperature. The average number densities were measured by LS and PTA methods for 2 days. As mentioned in our previous studies (Uchida et al., 2016a,b,c), the number density measured by the FFT method would be affected by the quenching process. Thus, we avoid the quantitative comparison between results obtained by different methods in the present study.

### C_3_H_8_-hydrate Formation and Evaluation of Promoting Effects

We used the same system as that in our previous study (Uchida et al., 2016a, 2020) for the C_3_H_8_-hydrate formation tests. Briefly, about 50 cm^3^ of liquid sample was set in a batch-type reaction vessel (inner volume: 232.2 cm^3^). To reduce formation of surface nanobubbles on the reaction vessel wall after introducing the liquid sample, the UFB-containing water was stored at room temperature for at least 1 h prior to its use in the experiments. The sample was free of visible bubbles. After the purge process, C_3_H_8_ gas was pressurized at a set value (about 0.45 MPa). The temperature of the vessel containing the sample was controlled by immersing in a cooling bath set at 273.9 ± 0.4 K. The C_3_H_8_-hydrate formation tests were started with a gentle agitation of about 300 rpm.

The promoting effect is defined as the decrease in length of the induction time of gas hydrate formation Δ*t* compared to the control condition (with pure water in the present study). The induction time is the time from when the temperature in the vessel reaches the equilibrium value to the time when the temperature of the vessel increases suddenly due to the exothermic process of hydrate formation. The latter time is also recognized by the sudden pressure drop due to the consumption of C_3_H_8_ gas. If the hydrate did not form by 50 h, we stopped the experiment and defined it “not formed.” As the nucleation process of gas hydrate is known to be stochastic, we evaluate the probability nucleation rate *P(t)* from 11 repeated experiments. The curve fitting was done by OriginPro (OriginLab, ver. 9.0J). The strength of the promoting effect <Δ*t*_*ind*_> is defined as the integration over time of *P*_*UFB*_*(t) – P*_*pw*_*(t)*, where *P*_*UFB*_*(t)* is the rate for the UFB-containing water, *P*_*pw*_*(t)* that for pure water.

For the statistical analysis, we estimate the significance using the Tukey-Kramer test (MS Excel 2010 and BellCurve) for at least 95% confidence (*p* < 0.05).

## Results and Discussion

### Distribution of UFBs in C_3_H_8_-hydrate Dissociated Water

**Figure 1:**
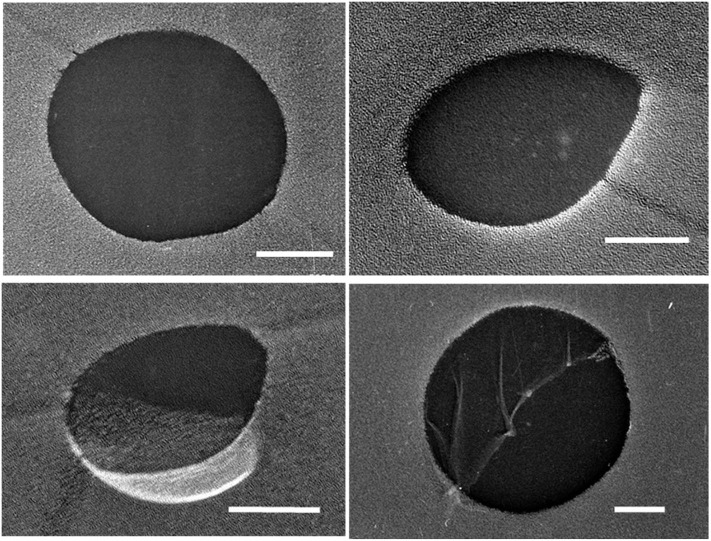
Typical TEM images of UFBs in C_3_H_8_-hydrate dissociation water by the freeze-fracture replica (FFT) method. Scale bars show 100 nm.

Some of the liquid sample used for the hydrate-formation experiment was set aside for analyzing its size distribution of UFBs by the LS method, the PTA method, and the FFT method. Figure 1 shows typical TEM images of C_3_H_8_ UFBs in the C_3_H_8_-hydrate dissociated water obtained by the FFT method. Consistent with this image, we found that most UFBs are spherical or oval, and that their size distributions had similarities to those observed in other hydrocarbon-gas UFBs (Uchida et al., 2016a,b).

We calculated the average particle size *D* and the number density *N* of C_3_H_8_ UFBs in each liquid sample. These quantities were calculated within 1 h of sample preparation and 1 day later. For example, results in Table 1 show for the LS measurements that UFBs over 300 nm in diameter had a number density of 10.7 (± 4.2) ×10^8^ mL^−1^ in the C_3_H_8_-hydrate dissociated water, but had the slightly lower concentration of 8.1 (± 2.7) ×10^8^ mL^−1^ in the C_3_H_8_-UFB-included water. These values are considered to be appropriate by the comparison to those obtained by the PTA method, although they are smaller than those obtained by other methods. Overall, there is little difference in number density between the UFB-included and the hydrate-dissociated samples. Thus, the C_3_H_8_ UFBs generated by the hydrate dissociation appear to roughly stabilize at the same number density as that prepared by the UFB generator. N.A. in Table 1 means that sufficient number of UFBs were not measured in liquid pure water.

**Table 1:** Average diameter *D* and number density *N* of UFBs in samples measured by LS, PTA, and FFT methods.

	***D* [nm]**	***N* [×10^**8**^ mL^**−1**^]**
C_3_H_8_-UFB-included water	> 300 (LS) 100 ± 20 → 124 ± 28 (PTA)	8.1 ± 2.7 → 6.5 ± 2.6 (LS) 0.77 ± 0.07 → 0.55 ± 0.11 (PTA)
C_3_H_8_-hydrate dissociated water	385 ± 283 → 746 ± 401 (FFT)* > 300 (LS) 133 ± 11 → 141 ± 14 (PTA)	6.4 ± 2.1 → 7.4 ± 3.2 (FFT) 10.7 ± 4.2 → 6.8 ± 4.1 (LS) 2.1 ± 1.3 → 1.7 ± 0.8 (PTA)*
	pH: 6.7	
Pure water	N.A.	N.A.

*Arrows show the change between the value within 1 h of sample preparation and that after about 24 h at room temperature. Asterisks mark those with a significant difference (p < 0.05). N.A. means that sufficient number of UFBs were not measured in liquid samples*.

This conclusion is consistent with findings from other hydrocarbon-gas hydrates (CH_4_: Uchida et al., 2016a; C_2_H_6_: Uchida et al., 2016b), although the number densities are larger than those from CO_2_-hydrate dissociated water (Uchida et al., 2020). In our previous study (Uchida et al., 2016c, 2020), we suggested that the UFB density might respond to the solubility and pH. Thus, we expect such similarity with other hydrocarbons because the solubility of C_3_H_8_ gas in water is similar to that of CH_4_ and because the pH value of the dissociated water is around seven (Table 1). In addition, the higher number density of UFBs in this case compared to the CO_2_-hydrate case is consistent with the higher pH conditions (Uchida et al., 2020).

**Figure 2:**
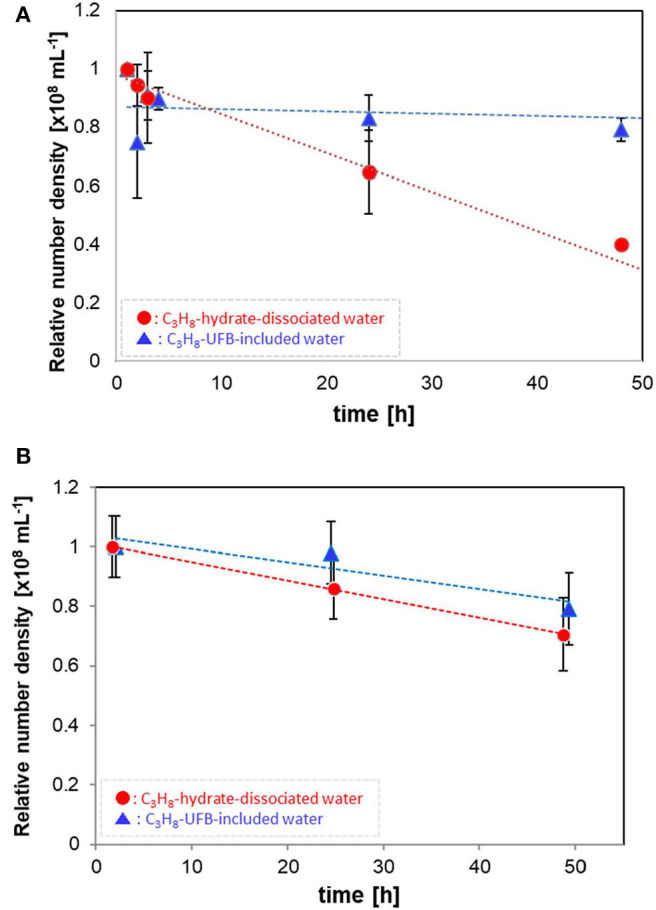
Number densities of C_3_H_8_ UFBs in the water samples normalized by the initial value. **(A)** Measured by the LS method (*D* > 300 nm). **(B)** Measured by the PTA method (*D* ~ 100 nm).

Figure 2 shows how the number densities decreased with time over 2 days. The values are normalized by the initial number density (averaged data, within 1 h of generation). Each error bar shows the standard deviation. The relatively large UFBs (larger than 300 nm) in the C_3_H_8_-hydrate dissociated water decrease in proportion to the storage time (Figure 2A), decreasing over 50% after 50 h. In contrast, these larger UFBs in the UFB-included water decrease initially by about 10%, within a few hours of generation, but then decreased much more slowly, decreasing another 10% over 50 h. Thus, after 50 h, the residual ratio is about 0.8, about twice that of the C_3_H_8_-hydrate dissociated water. Assuming a linear decrease with time, the decrease rates of UFBs in the UFB-included water and in the C_3_H_8_-hydrate dissociated water are about –0.08 × 10^6^ mL^−1^ h^−1^ and –1.33 × 10^6^ mL^−1^ h^−1^, respectively. That is, the difference in the decrease rates is over an order of magnitude.

For the smaller UFBs, the number densities decrease as shown in Figure 2B. Despite the initial number densities differing significantly (Table 1), the residual ratios of UFBs around 100 nm in diameter are nearly equal after about 50 h. Specifically, their linear rates of decrease are about –0.50 × 10^6^ mL^−1^ h^−1^ for the UFB-included samples and –0.62 × 10^6^ mL^−1^ h^−1^ for the hydrate-dissociated samples. Therefore, for both the larger and smaller UFBs, the number in the hydrate-dissociated water tends to decrease faster than that in the UFB-included water, at least over the size range observed here.

**Figure 3:**
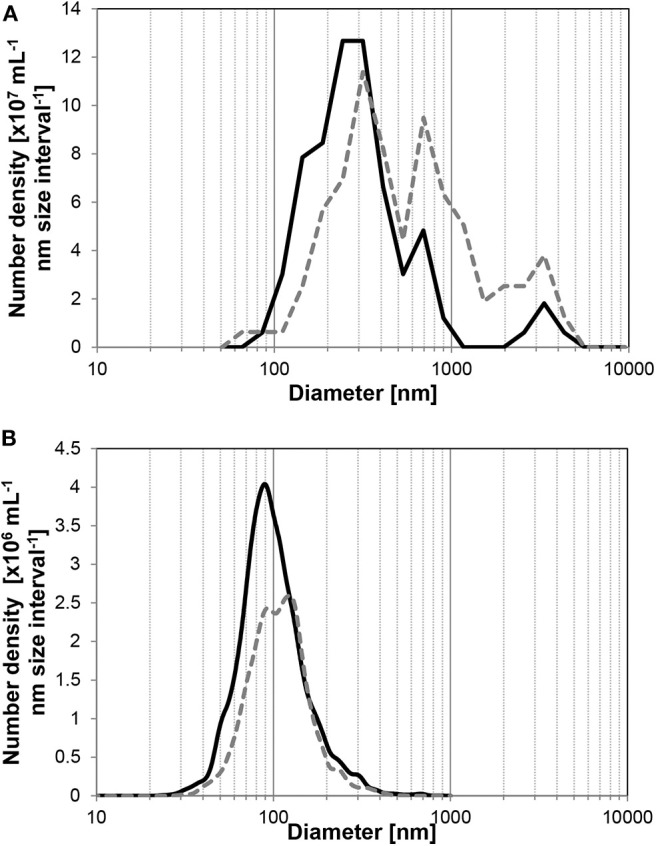
Average size distributions of UFBs in C_3_H_8_-hydrate dissociated water. Solid line is the initial distribution, dashed line is that after 1 day. **(A)** By FFT observations (*n* > 4). **(B)** By PTA measurements (*n* > 6).

The size distribution of UFBs also changed with time. To observe the size distribution of UFBs in wider range, we must combine the different measurement methods here. As shown in Figure 3, the FFT measurement covers the larger UFBs whereas the PTA measurement covers smaller ones which slightly overlaps at the range about 100 nm. The FFT measurements of larger UFBs in the hydrate-dissociated samples in Figure 3A shows that the distribution shifts to larger sizes over time. Similarly, the size distribution from the PTA measurements show a shift to larger sizes (Figure 3B). This shift is small, and arises from a preferential decrease in the UFBs smaller than 100 nm. These data suggest that the initial distribution has a large distribution of sizes, from several tens of nanometers to several micrometers, but that after 1 day or more, the average value increases due to the disappearance of small UFBs or the growth of UFBs into micro-bubbles. These trends in average diameter and number density suggest Ostwald ripening, in which small UFBs dissolved and large UFBs grew, with the largest UFBs disappearing during the storage period due to their increase in buoyancy. But regardless of these processes, the number density remained of order 10^8^ mL^−1^ in the C_3_H_8_-hydrate dissociated water during a 2-day storage period.

For the larger UFBs (over 300 nm diameter), the difference in lifespans (residual ratio) between that in the UFB-included water and that in the C_3_H_8_-hydrate dissociated water is likely due to the difference in the UFB-generation methods. As the UFB-included water was prepared with 1-h aeration during the UFB generation, the C_3_H_8_ concentration in the water should be sufficiently saturated. However, the C_3_H_8_-hydrate dissociated water is prepared by dissolving several crystalline pieces in pure water. Thus, the solution might not initially be saturated. UFBs are stable in water supersaturated with the source gas (Uchida et al., 2016c). Therefore, the residual ratio of UFBs in the C_3_H_8_-hydrate dissociated water would be lower than in C_3_H_8_-UFB-included water because most of the UFBs initially formed during hydrate dissociation soon dissolve into the water. UFBs larger than 300 nm tend to dissolve preferentially in the smaller supersaturated solution (e.g., in C_3_H_8_-hydrate dissociated water), whereas UFBs smaller than 100 nm preferentially dissolve in the sufficiently supersaturated solution (e.g., in C_3_H_8_-UFB included water).

The total number density of C_3_H_8_ UFBs in the solution in which C_3_H_8_ hydrate has just dissociated is estimated to be of order 10^9^~10^10^ mL^−1^. So, the use of C_3_H_8_-hydrate dissociated water, as done in other memory-effect experiments (such as (Zeng et al., 2006)), should have a sufficient initial supply of C_3_H_8_. The lifespan of UFBs in such dissociated water should be as long as that observed in the UFB-included water prepared by the UFB generator.

### Induction Time Measurements of C_3_H_8_ Hydrates

**Figure 4:**
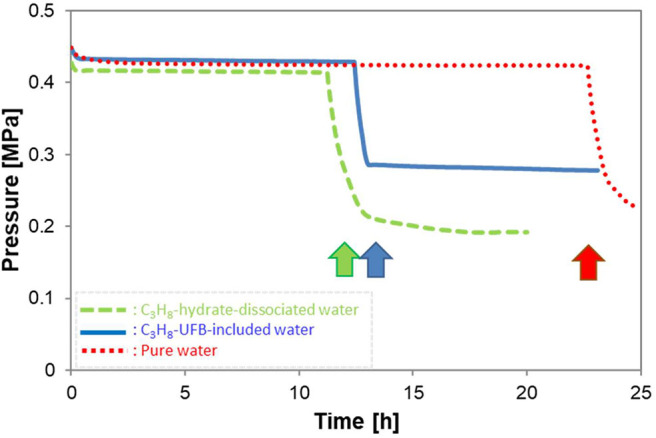
Typical pressure profiles during hydrate formation with three kinds of solutions. Arrows show the hydrate formation point, giving the induction time.

Figure 4 shows typical pressure profiles in the vessel with formation of C_3_H_8_ hydrate under the conditions of *P* = 0.45 MPa and *T* = 273.9 K. All three types of liquid samples are shown. After C_3_H_8_ gas was introduced into the vessel, its pressure decreased slightly due to the temperature drop from room temperature. In the figure, time zero is when the temperature and pressure of the vessel reached the equilibrium ones (about 278 K at 0.45 MPa). Thus, the subsequent pressure drop indicates C_3_H_8_-hydrate formation (shown by arrows), so the time of this sudden drop in pressure is the induction time. Simultaneously with the pressure drop, the temperature rose. But of every 8 experiments with hydrate formation, about 3 others did not produce hydrate within 50 h. When the latter occurred, we counted it as “non-generation.”

Instead of the tens of minutes induction time of other gas hydrates (C_2_H_6_ hydrate: (Uchida et al., 2016b) and CO_2_ hydrate: (Uchida et al., 2020)), the C_3_H_8_-hydrate formation required tens of hours (Figure 4). The longer induction time indicates that C_3_H_8_ hydrate has a larger energy barrier for crystal formation than other gas hydrates. As a consequence, the promoting effect for C_3_H_8_ hydrate has greater importance for controlling the hydrate-formation processes. Figure 4 also shows induction times are nearly halved in the C_3_H_8_-hydrate dissociated water and C_3_H_8_-UFB-included water over that in pure water. This result shows a strong promoting effect from using C_3_H_8_ UFBs.

**Figure 5:**
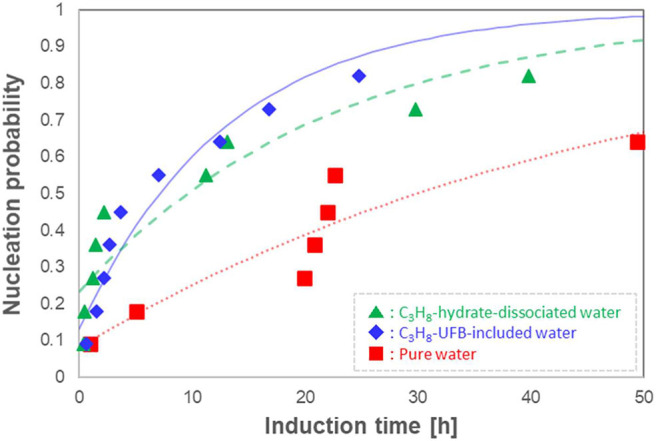
Nucleation probability of C_3_H_8_ hydrate vs. induction time (*n* = 11). Each curve is a fit from Equation (1).

Given the stochastic behavior of crystal formation, we repeated the induction-time measurements 11 times under the same conditions, determining the probability distributions as done in Sowa and Maeda (2015). We show the resulting induction time series in Figure 5 as probability nucleation rate curves. This figure shows that both nucleation rate curves of the C_3_H_8_-hydrate dissociated water (▲) and of the C_3_H_8_-UFB-included water (♦) are shifted to shorter induction times than that of pure water (■). Thus, both types of liquid samples containing C_3_H_8_ UFBs exhibit a promoting effect and follow nearly identical curves. In addition, the formation probability within 50 h was 0.8 in both C_3_H_8_-UFB containing solutions, about 1.3 times that found for pure water.

Concerning the relatively long induction times of C_3_H_8_-hydrate, the difficulty of formation had been explained by the labile-cluster nucleation hypothesis and assumed that it was in the “difficulty of producing 5^12^6^4^ cavities” (Christiansen and Sloan, 1994; Sloan and Koh, 2007). Thus, if the memory effect of C_3_H_8_ hydrate is explained using the water structuring theory, the induction time with C_3_H_8_-hydrate dissociated water should be significantly shorter than that with UFB-included water, as the latter has not experienced any hydrate structure. However, the results show that both samples exhibit a similar promoting effect. We conclude that the presence of UFBs, which is a common feature of both aqueous solutions, had a dominant effect on the exhibition of the memory effect of C_3_H_8_ hydrate. Thus, as we found earlier (Uchida et al., 2016a,b, 2020), this conclusion supports the guest dissolution hypothesis for the memory effect on C_3_H_8_ hydrates, not the water structuring hypothesis.

We now analyze the promoting effect of C_3_H_8_ hydrate more quantitatively. To compare the fitting parameters with those obtained in previous studies (Takeya et al., 2000; Uchida et al., 2016b, 2020), we fit the normalized nucleation probability *P(t)* curves of Figure 5 to

(1) $$\begin{array}{l} P\left(t\right)=1-\exp\left[-J\left(t-\tau_{0}\right)\right], \end{array}$$

where *J* is the nucleation rate and τ_0_ the offset time. Table 2 shows the resulting fits. The resulting values of τ_0_ are small and negative, indicating that most of the nucleation occurs at an early stage compared to other long induction times. The fits in Table 2 also show that the nucleation rate *J* is larger in both C_3_H_8_-hydrate dissociated water and UFB-included water than that in pure water. However, compared to the rate increase by factors of 100 and 110 for C_2_H_6_ hydrate (Uchida et al., 2016b), these increases are only factors of about 2.2 times and 3.8 times, respectively, compared to that with pure water.

**Table 2:** Nucleation probability parameters (Equation 1).

**Sample**	**τ_0_ [h]**	***J* [×10^**−2**^ h^**−1**^]**	**<Δ*t*_*ind*_> [h]**
C_3_H_8_-UFB-included water	−1.8	7.78 ± 1.09	14.1
C_3_H_8_-hydrate dissociated water	−5.9	4.48 ± 1.00	16.9
Pure water	−4.2	2.03 ± 0.41	—

To quantify the promoting effect, we estimate the expected induction time <Δ*t*_*ind*_> following the method of Sowa and Maeda (2015) and Uchida et al. (2016b). We compare the difference of areas below the nucleation probability curves between the test water and pure water (Figure 5). The resulting values give the magnitude on the promoting effect of C_3_H_8_-hydrate dissociated water and C_3_H_8_-UFB-included water. As shown in the last column of Table 2, these two aqueous solutions have nearly the same value, which is consistent with the results obtained for C_2_H_6_ hydrate (Uchida et al., 2016b). The reason why the data and the curve do not fit well is considered to be mainly the small number of data. The additional number of experiments under the same condition would provide better solution in the future studies.

The above comparisons show that the UFBs exhibit the memory effect in C_3_H_8_ hydrate. Given that the nucleation of C_3_H_8_ hydrate is much more difficult than those of other gases, the exhibition of a promoting effect can be significant. For example, for C_2_H_6_ hydrate, the time at which the nucleation probability reaches 1 is about 1.2 h in pure water (Uchida et al., 2016a) and about 0.7 h for CO_2_ hydrate (Uchida et al., 2020), whereas for C_3_H_8_ hydrate, the probability of formation was as low as 0.6 even for 50 h. With the promoting effect of UFBs, the time for nucleation of C_2_H_6_ hydrate is shorter by only about 15 min (Uchida et al., 2016a), whereas for C_3_H_8_ hydrate the time was shorter by more than 20 h. In this way, the use of UFB-containing water is a promising way to promote those gas hydrates that are difficult to nucleate.

### Roles of UFBs on the Promoting Effects of C_3_H_8_ Hydrates

The stability measurements indicate that the UFBs remained in high concentration (~10^8^ mL^−1^) even after 50 h and stored at room temperature, we argue that they have a role in the promotion effect on C_3_H_8_ hydrate.

As the nucleation of gas hydrates occurs preferentially at the gas-liquid interface (Sloan and Koh, 2007), the induction time should be shorter in water with a much larger interface area, that is, one containing many UFBs. The nucleation probability in Figure 5 shows that C_3_H_8_ hydrates are formed at a higher rate in the narrow Δ*t* range in the UFB-containing waters. This is associated with the increase of value of *J*. The increase of *J* is also observed in other gas hydrate systems with UFBs (Uchida et al., 2016a, 2020). Thus, we assume that the gas-hydrate formation involves heterogeneous nucleation on the gas-liquid interface as argued previously.

**Figure 6:**
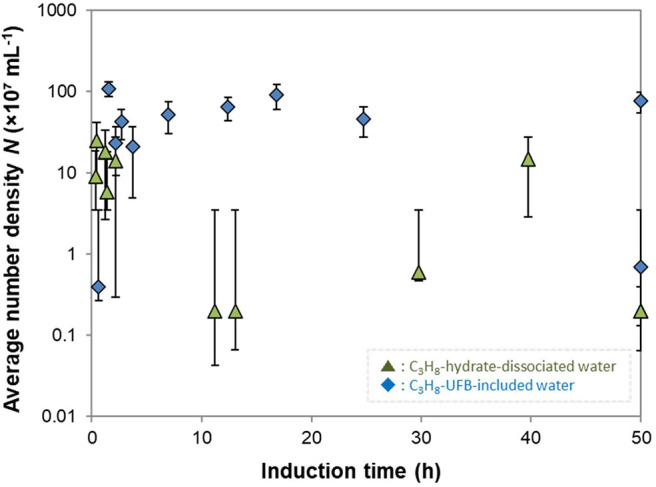
Average number density of UFBs *N* and induction times. Error bars show the standard deviation of measured number densities (*n* > 16).

To further investigate the roles of UFBs on hydrate formation, we measured the number density of UFBs in the aqueous solutions by the LS method prior to the formation experiments. Figure 6 shows the dependence of the induction time on the number density of larger UFBs (>300 nm). This figure shows that the aqueous solutions contained UFBs with a number density *N* of 10^6^ to 10^9^ mL^−1^. This figure also shows that the induction time does not clearly depend on *N*. This result suggests that the hydrate-formation process is not limited by the total area of the gas-liquid interface from the population of UFBs. Thus, the gas-liquid interface appears crucial for nucleation, but its total area is not a key parameter. An explanation for this behavior was proposed by Lipenkov (2000) who suggested that the hydrate nucleation would preferentially occur at a certain size of bubble. In his investigation of air-hydrate distributions in the ice matrix retrieved from a deep ice sheet in Antarctica, he proposed a nucleation process in which air-hydrate crystals transformed from air bubbles smaller than a critical size. Testing this hypothesis requires both a greater number of hydrate formation tests under the same conditions and the actual size distribution of UFBs in each liquid sample over the diameter range from 10^−9^ to 10^−5^ m. However, both of them are unfortunately difficult at present. As suggested by our results in Figure 3, it is still difficult to obtain the combined size distribution of UFBs obtained by FFT observations and by PTA measurements. Further quantitative investigations are needed.

## Conclusion

To help improve the technology for producing gas hydrate, we investigated the promoting mechanism for the memory effect on propane (C_3_H_8_) hydrate. C_3_H_8_ is the main component of LPG, and an important component of natural gas, so its control technology is very important. However, C_3_H_8_ hydrate is difficult to form from pure water and pure C_3_H_8_ gas. This difficulty was reconfirmed in the present study. In particular, the nucleation probability within 50 h was about 0.6, much lower than that found previously for ethane (C_2_H_6_) hydrates. Therefore, the development of the formation-promotion technology on C_3_H_8_ hydrate is important.

We found that a key factor in the promoting effect is the presence of ultra-fine bubbles (UFBs). As had been found previously from dissociation of CH_4_ and C_2_H_6_ hydrates, the dissociation of C_3_H_8_-hydrate produced a similar amount of UFBs. Thus, UFBs have been found in both the dissociation of sI-type hydrates (CH_4_ and C_2_H_6_) and sII-type hydrate (C_3_*H*_8_). Concerning these UFBs, their number density tended to decrease with time, likely controlled by the saturation condition with guest gas in water. However, the fraction remaining within 50 h was at least 0.4, with more than 10^7^ mL^−1^ remaining in water after 50 h.

We compared the memory effect on C_3_H_8_ hydrates between two C_3_H_8_-UFB containing waters, specifically, C_3_H_8_-hydrate dissociated water and C_3_H_8_-UFB-included water prepared by an UFB generator. Based on 11 experiments with C_3_H_8_-hydrate formation, we found that the nucleation probability within 50 h was 1.3 times larger than that of the case with pure water, and that the induction time was shortened by nearly half. Therefore, we confirmed that UFB-containing water promoted the formation of C_3_H_8_ hydrates, with the two types of UFB-containing water giving nearly the same nucleation probability curve. We argued that this similarity does not support the idea that the promotion is due to a hydrate-memory structure in the water. In addition, we found little correlation between the initial UFB number density and nucleation probability. Therefore, we argue that the memory effect of gas hydrates arises from the existence of guest-gas UFBs, which are mainly playing a role as the guest-gas supplying source, thus supporting the gas dissolution hypothesis.

## Data Availability Statement

The raw data supporting the conclusions of this article will be made available by the authors, without undue reservation.

## Author Contributions

TU, KY, and KG contributed conception and design of the study. AS performed the statistical analysis. TU wrote the first draft of the manuscript. TU, HM, and RS wrote sections of the manuscript. All authors contributed to manuscript revision, read and approved the submitted version.

## Conflict of Interest

The authors declare that the research was conducted in the absence of any commercial or financial relationships that could be construed as a potential conflict of interest.

## Figures and Tables

**Table:** 

*D*	Average diameter of UFBs
*J*	Nucleation frequency
*N*	Number density of UFBs
*τ_0_*	Offset time
*<Δt_*ind*_>*	Expected induction time
*P*(*t*)	Normalized nucleation probability by time *t*

## Abbreviations

UFB: ultra-fine bubble (sub-micron sized)
FFT: freeze-fractured replica observation via transmission electron microscope
LS: laser-light scattering
PTA: particle tracking analysis.

## References

[B1] Bagherzadeh S. A. Alavi S. Ripmeester J. A. Englezos P. (2015). Formation of methane nano-bubbles during hydrate decomposition and their effect on hydrate growth. J. Chem. Phys. 142:214701. 10.1063/1.4920971 26049510

[B2] Buchanan P. Soper A. K. Thompson H. Creek J. L. Hubson G. Koh C. A. (2005). Search for memory effects in methane hydrate: structure of water before hydrate formation and after hydrate decomposition. J. Chem. Phys. 123:164507. 10.1063/1.2074927 16268712

[B3] Christiansen R. L. Sloan E. D. Jr. (1994). Mechanisms and kinetics of hydrate formation. Ann. NY Acad. Sci. 715, 283–305. 10.1111/j.1749-6632.1994.tb38841.x

[B4] Giavarini C. Maccioni F. Santarelli M. L. (2003). Formation kinetics of propane hydrates. Ind. Eng. Chem. Res. 42, 1517–1521. 10.1021/ie0207764

[B5] Gudmundsson J. Borrehaug A. (1996). Frozen hydrate for transport of natural gas. Proc. ICGH 2, 415–422.

[B6] Horiguchi K. Watanabe S. Moriya H. Nakai S. Yoshimitsu A. Taoda A. (2011). Completion of natural gas hydrate (NGH) overland transportation demo project, in Proceedings of 7th International Conference (Edinburgh: Natural Gas Hydrates), 17–21.

[B7] Hwang M. J. Wright D. A. Kapur A. Holder G. D. (1990). An experimental study of crystallization and crystal growth of methane hydrates from melting ice. J. Inclusion Phenom. Molecul. Recogn. Chem. 8, 103–116. 10.1007/BF01131291

[B8] Ida H. Kohda K. (2004). Highly Efficient Natural Gas Hydrate Production Technology. JFE Technical. Report No. 6, 76–80 (in Japanese with English abstract).

[B9] ISO 20408-1:2017. (2017). Fine Bubble Technology - General Principles for Usage and Measurement of Fine Bubbles - Part 1: Terminology. Available online at: https://www.iso.org/standard/68187.html?browse=tc (accessed Febuary 27, 2020).

[B10] Kvenvolden K. A. (1988). Methane hydrate - a major reservoir of carbon in the shallow geosphere? Chem. Geol. 71, 41–51. 10.1016/0009-2541(88)90104-0

[B11] Lipenkov V. Y. (2000). Air bubbles and air-hydrate crystals in the vostok ice core, in Physics of Ice Core Records. ed Hondoh T. (Sapporo: Hokkaido Univ. Press), 327–358.

[B12] Masuda Y. Uchida T. Nagakubo S. Satoh M. (2016). Methane hydrates, in Fossil Fuels: Current Status and Future Directions, World Scientific Series in Current Energy Issues, ed Crawley G. M. (Singapore: World Scientific Pub. Co. Pte. Ltd.), 289–327. 10.1142/9789814699983_0010

[B13] Mimachi H. Takeya S. Yoneyama A. Hyodo K. Takeda T. Gotoh Y. (2014). Natural gas storage and transportation within gas hydrate of smaller particle: size dependence of self-preservation phenomenon of natural gas hydrate. Chem. Eng. Sci. 118, 208–213. 10.1016/j.ces.2014.07.050

[B14] Ohmura R. Ogawa M. Yasuoka K. Mori Y. H. (2003). Statistical study of clathrate-hydrate nucleation in a water/hydrochlorofluorocarbon system: search for the nature of the “memory effect”. J. Phys. Chem. B 107, 5289–5293. 10.1021/jp027094e

[B15] Oshita S. Uchida T. (2013). Basic Characterization of nanobubbles and its potential applications, in Bio-Nanotechnology: a Revolution in Biomedical Sciences, and Human Health. eds Bagchi D. Bagchi M. Moriyama H. Shahidi F. (West Sussex, UK: John Wiley and Sons, Ltd.), 506–516. 10.1002/9781118451915.ch29

[B16] Parent J. S. Bishnoi P. R. (1996). Investigation into the nucleation behavior of methane gas hydrates. Chem. Eng. Commun. 144, 51–64. 10.1080/00986449608936444

[B17] Ripmeester J. A. Alavi S. (2016). Some current challenges in clathrate hydrate science: nucleation, decomposition and the memory effect. Curr. Opin. Solid State Mater. Sci. 20, 344–351. 10.1016/j.cossms.2016.03.005

[B18] Rodger P. M. (2000). Methane hydrate, melting and memory. Ann. N.Y. Acad. Sci. 912, 474–482. 10.1111/j.1749-6632.2000.tb06802.x

[B19] Seddon J. R. T. Lohse D. Ducker W. A. Craig V. S. J. (2012). A deliberation on nanobubbles at surfaces and in bulk. ChemPhysChem 13, 2179–2187. 10.1002/cphc.201100900 22378608

[B20] Sefidroodi H. Abrahamsen E. Kelland M. A. (2013). Investigation into the strength and source of the memory effect for cyclopentane hydrate. Chem. Eng. Sci. 87, 133–140. 10.1016/j.ces.2012.10.018

[B21] Sloan E. D. (2004). Fundamental principles and applications of natural gas hydrates. Nature 426, 353–363. 10.1038/nature02135 14628065

[B22] Sloan E. D. Koh C. A. (2007). Clathrate Hydrate of Natural Gases, 3rd Edn Boca Raton, FL: CRC Press 10.1201/9781420008494

[B23] Sowa B. Maeda N. (2015). Statistical study of the memory effect in model natural gas hydrate systems. J. Phys. Chem. A 119, 10784–10790. 10.1021/acs.jpca.5b07308 26506447

[B24] Takahashi M. (2005). ζ potential of microbubbles in aqueous solutions: electrical properties of the gas-water interface. J. Phys. Chem. B 190, 21858–21864. 10.1021/jp0445270 16853839

[B25] Takeya S. Hori A. Hondoh T. Uchida T. (2000). Freezing-memory effect of water on nucleation of CO2 hydrate crystals. J. Phys. Chem. B 104, 4164–4168. 10.1021/jp993759+

[B26] Takeya S. Mimachi H. Murayama T. (2018). Methane storage in water frameworks: self-preservation of methane hydrate pellets formed from NaCl solutions. Appl. Energy 230, 86–93. 10.1016/j.apenergy.2018.08.015

[B27] The Chemical Society of Japan (2004). Kagaku-binran Handbook of Chemistry, 5th Edn Tokyo: Maruzen Co. Ltd 144–149.

[B28] Uchida T. Liu S. Enari M. Oshita S. Yamazaki K. Gohara K. (2016c). Effect of NaCl on the lifetime of micro- and nanobubbles. Nanomaterials 6:31. 10.3390/nano6020031 28344288 PMC5302484

[B29] Uchida T. Miyoshi H. Yamazaki K. Gohara K. (2020). Effects of ultra-fine bubbles on exhibiting the memory effect, in Proceedings of 10th Interenational Conference (Singapore: Natural Gas Hydrates).

[B30] Uchida T. Yamazaki K. Gohara K. (2016a). Generation of micro- and nano-bubbles in water by dissociation of gas hydrates. Korean J. Chem. Eng. 33, 1749–1755. 10.1007/s11814-016-0032-7

[B31] Uchida T. Yamazaki K. Gohara K. (2016b). Gas nano-bubbles as nucleation acceleration in the gas-hydrate memory effect. J. Phys. Chem. C 120, 26620–26629. 10.1021/acs.jpcc.6b07995

[B32] Uchida T. Yamazaki K. Gohara K. (2017). Generation of gas nano-bubbles by gas hydrate dissociation and its effect on the memory effect, in Proceedings of 10th Interenational Conference (Denver, CL: Natural Gas Hydrates).

[B33] Yagasaki T. Matsumoto M. Andoh Y. Okazaki S. Tanaka H. (2014). Effect of bubble formation on the dissociation of methane hydrate in water: a molecular dynamic study. J. Phys. Chem. B 118, 1900–1906. 10.1021/jp412692d 24471937

[B34] Zeng H. Moudrakovski I. L. Ripmeester J. A. Walker V. K. (2006). Effect of antifreeze protein on nucleation, growth and memory of gas hydrates. AIChE J. 52, 3304–3309. 10.1002/aic.10929

